# Women’s empowerment and remaining within ideal family size in the East African Community

**DOI:** 10.1186/s12978-026-02412-y

**Published:** 2026-07-24

**Authors:** Ange Chanelle Akimana, Zoran Bukumiric, Didace Kana, Dejana Vukovic

**Affiliations:** 1 Faculty of Medicine, University of Belgrade, Belgrade, 11000 Serbia; 2 Institute for Medical Statistics and Informatics, Faculty of Medicine, University of Belgrade, Belgrade, Serbia; 3 Department of Clinical Research, Faculty of Health Sciences, University of Southern Denmark, Odense, Denmark; 4 Gitega Regional Hospital- Hôpital Régional de Gitega, Gitega, Burundi; 5 Institute of Social Medicine, Faculty of Medicine, University of Belgrade, Belgrade, 11000 Serbia

**Keywords:** Fertility outcomes, Family size, DHS, SWPER index, Sub-Saharan Africa, Gender equality

## Abstract

**Background:**

One in every 27 people worldwide belongs to the East African Community, a region with shared cultural traits and free movement. Despite recent declines, the fertility rates in this Community remain high. This study assesses the link between women’s empowerment and remaining within the woman’s ideal family size, considering the women’s perceptions of their husbands’ desired family size.

**Methods:**

This study used Demographic and Health Survey datasets of Burundi (2015–2016), The Democratic Republic of the Congo (2013–2014), Kenya (2022), Rwanda (2019), Tanzania (2022), and Uganda (2016). A total of 5855 married or cohabiting women aged 35–49 who perceive that their husband wants more children than they do were included in this study. IBM SPSS Statistics 24 (SPSS: Statistical Package for the Social Sciences) was employed for data analysis. The Survey-based Women’s empowerment (SWPER) index, across its three domains (attitude toward violence, social independence, and decision-making) was adopted to evaluate empowerment levels. Binary logistic regression was implemented to examine the association between the domains and the outcome variable, which indicates whether a woman has remained within her ideal number of children. Additionally, the link between potential confounders and the outcome variable was assessed. Univariable and multivariable models including country fixed effects were estimated. Sensitivity analyses excluded women below their ideal number. Variables showing p-values < 0.05 were considered statistically significant.

**Results:**

Overall, 63.3% of women were within their stated ideal number. Most women had medium levels of empowerment across all the index domains. Empowerment in the domains of social independence and decision-making was significantly associated with remaining within the woman’s goal (Adjusted Odds Ratios: 1.41 (95% CI 1.28–1.56) and 0.80 (95% CI 0.72–0.89), respectively). From control variables, woman’s age, household size, and ability to read a sentence showed significant association with the outcome. Only social independence was positively linked to the outcome. Attitude toward violence showed no significant association with the dependent variable in the univariable model.

**Conclusions:**

In settings where gender power imbalances may persist, strengthening women’s autonomy and their reproductive health agency with a focus on factors that promote social independence empowerment is needed in the East African Community.

**Supplementary Information:**

The online version contains supplementary material available at https://doi.org/10.1186/s12978-026-02412-y.

## Abstract (plain language summary)

Despite the decline in the total fertility rates, five of eight member states of the East African Community are ranked among the top ten high-fertility countries globally. This study aims to assess the link between women’s empowerment and remaining within their ideal number of children, taking into account the women’s perceptions of their husbands’ desired family size.

We used nationally representative data from the most recent demographic and health surveys of six member states of the East African Community. We included 5855 married or cohabiting women aged 35 to 49, who perceive that their husbands desire more children than they do. To assess the women’s empowerment levels, we used an index made up of three domains: attitude toward violence, social independence, and decision-making. We analyzed the association between remaining within the ideal number of children and the index’s domains while controlling for potential confounders.

Sixty-three and three percent of all the women were able to remain within their desired fertility. The results showed that the more a woman is empowered in the domain of social independence, the more she can remain within her desired family size. On the contrary, the higher the woman’s empowerment level in the domain of decision-making, the less the chances for the woman to reach her goal. The domain of attitude toward violence was not associated with remaining within the number of children.

Promotion of factors that enhance social independence would be one big step for women in the East African community.

**Supplementary Information:**

The online version contains supplementary material available at https://doi.org/10.1186/s12978-026-02412-y.

## Background

Despite a progressive decline in total fertility rates, the global population continues to rise over time [1, 2]. Significant population imbalances are noticeable between countries and continents and within individual continents [3]. Starting from its peak in 1965, when the world population rose by 2.24%, the percentages of annual population change have generally declined to a level of 0.83% in 2022 (the lowest to date) and 0.90% in 2024 [1]. The total fertility rate has dropped from about five children per woman until the late 1960s to an average of fewer than 2.5 children today in 2025 [4]. Despite this, the United Nations projects that the world population will rise to 9.7 billion by 2050 and 10.4 billion by 2100 [2, 3].

Fertility patterns vary across continents, countries, and regions. Opposite to the majority of developed countries, most developing nations have high fertility rates, often surpassing the replacement level. The latter is the point at which a population replaces itself from one generation to the next and was estimated at 2.3 births in 2023 and is projected to decline to 1.8 births by 2100 [3]. Europe and East Asia demonstrates below-replacement fertility, with countries like Singapore and Ukraine reporting fertility levels below one birth per woman according to the 2024 reports [5]. Latin America has seen dramatic declines—Brazil’s and Chile’s fertility for instance fell from more than five children to 1.7children between 1960 and 2025 [6, 7]. South Asia shows uneven progress, with Bangladesh reaching replacement level while Afghanistan remains the highest in the region with a fertility rate of approximately 4.7 children per woman in 2025 [6]. Africa itself displays sub-regional variation. North African countries (Morocco, Tunisia, Algeria) have achieved fertility declines to near-replacement levels whereas sub-Saharan countries like Chad and Somalia report more than six births per woman [5]. The East African Community (EAC), which makes up our study setting, comprises seven eastern and one central African country [8]. As of May 2025, it had a population of 331.1 million [9], meaning that roughly one in every 27 people worldwide is from the EAC. Total fertility rates have declined in the EAC as in the rest of the world [3], but five countries belonging to this community are classified among the top ten countries with the highest fertility rates [10].

Most countries where fertility rates are above or below the replacement level have already introduced policies to either curb or encourage fertility [11]. These policy responses are rooted in demographic theories that relate fertility transitions to the achievement of multiple Sustainable Development Goals (SDGs) [12–16]. The demographic transition theory, which addresses two fundamental demographic dynamics: fertility, and mortality; has linked changes in fertility rates to shifts in the ideal number of children [12]. A central element in fertility discussions is women’s reproductive agency. Undeniably, women have a crucial role in reproduction. However, in many low-income contexts, women are seen as childbearing machines dominated by patriarchal systems and face pressure to conceive within marriage [17–19] These patriarchal structures are reinforced by broader socio-cultural factors that shape a couple’s desired family size, ranging from their personal choices, societal expectations, religious beliefs, and childhood family environment [20–23]. Additionally, in some settings, there is a culturally expected number of children a woman should have, and having children below that number can lead to social disapproval. A woman may be considered truly married after bearing at least one child, and widows of childless men risk being sent away. Consequently, many women rush to conceive immediately after marriage, regardless of financial or mental readiness, despite evidence linking early childbearing to exceeding ideal family size [18, 24–27].

Women, especially the less empowered ones who lack the agency to negotiate and maintain control over the reproductive outcomes may feel pressure to conform to the wishes of their spouses or society in case their fertility goal diverges their husbands’ [27–29]. Research has shown that women who perceive that their husbands want more children than them or do not know their husbands’ fertility desires have decided to practice covert contraception use [30–32]. In contrast, empowering and educating women has been linked to the ability to negotiate and make better decisions in their reproductive lives, such as reaching their ideal number of children, using contraception, and spacing births, among others [33–37].

In addition to cultural expectations, patriarchal norms, and religious beliefs that influence couples’ fertility preferences in the EAC; child labor is another critical factor when discussing ideal family size in the EAC. A child is expected to help with household chores at a certain age. In many rural communities, older children are expected to care for their younger siblings and contribute to the family economy [35, 38, 39]. Moreover, the East African region reports some of the highest rates of child labor globally, and South Soudan ranks first worldwide [40].

Studies have previously analyzed the relationship between women’s empowerment and the ideal number of children in Africa and developing countries [33–37, 41, 42]. However, based on our literature review, most were conducted in West African countries or Asian and South American developing countries. This study aims to answer the question: Is women’s empowerment associated with remaining within ideal family size among women who believe their husbands desire more children than they do? To our knowledge, no studies have examined whether empowered women are more likely to not exceed their fertility goals despite perceived spousal pressure for higher fertility. This region-specific study uses a well-established empowerment index that assesses empowerment on a broader level [43, 44 ].

### Conceptual framework

Using well-established literature as a basis [33–37], we propose that women’s empowerment, as measured through the domains of the SWPER index used in this study (attitude toward violence, social independence, and decision-making), is positively associated with remaining within a woman’s ideal family size in the EAC. Each domain of the index itself has documented links to reproductive health [44]. However, given the multidimensional nature of the concept of empowerment, its relationship with fertility outcomes may not be uniform across domains. While the social independence reflecting education, access to information, and exposure to media may equip women with the knowledge and resources needed to turn their fertility intentions into actual fertility outcomes, decision making empowerment maybe more complex. As above-mentioned in the background, in certain high fertility settings, childbearing grants social status, hence a woman’s empowerment could be a result of having had many children. Furthermore, although a woman who rejects wife-beating is likely to have stronger personal agency which may enhance her ability to act on her fertility preferences and remain within her desired number of children, this is not a golden rule as empowerment is multidimensional and may operate differently across contexts. Our analysis therefore examines each domain separately, allowing for the possibility of domain-specific effects. 

## Methods

### Data source and study participants

This retrospective study uses secondary data from the Demographic and Health Surveys (DHS) [45]. DHS are nationally representative datasets collected across developing countries, with the primary goal of supporting data-based decisions and monitoring their progress toward the SDGs. They are conducted in five-year phases with iterative improvements. Using different models, various questions are prepared for women, children, husbands, and households.

**Fig. 1:**
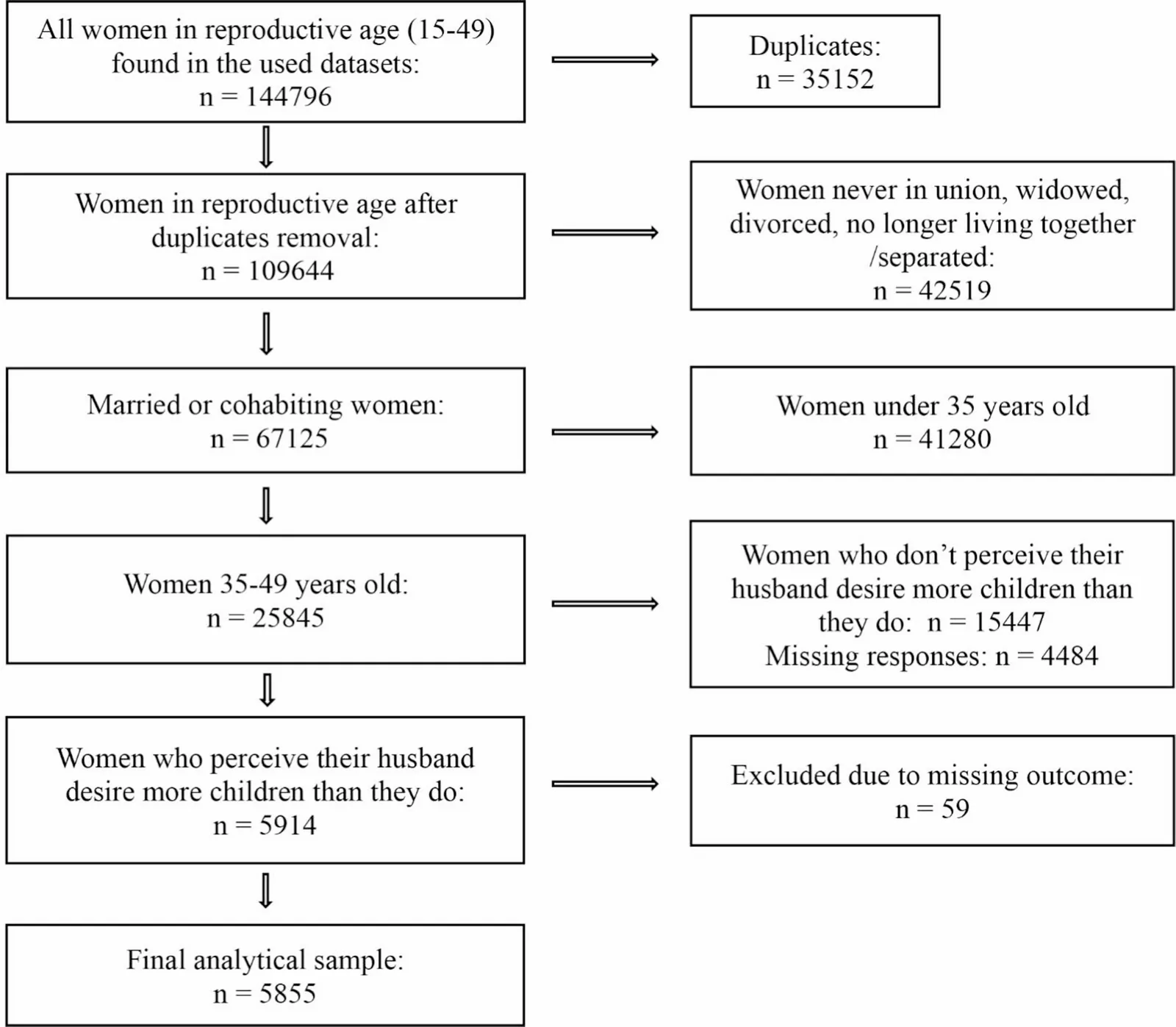
Participant flow diagram

Our study uses the woman’s questionnaire, which comprises information about women in the reproductive age [15–49]. Given that empowerment is our primary independent variable and that questions about empowerment were addressed to only women who are married or cohabiting, and due to the aim of our study, we focus on women 35–49 years old, as this cohort has been used in several demographic literatures, especially studies using DHS data for analyzing completed fertility [34, 36, 37]. Also, we selected only women who reported perceiving that their husbands desire more children than they do. Perceiving a spouse’s desire for more children represents a potential source of internalized pressure that can influence a woman’s reproductive decisions. By focusing on this subgroup of women, we will be able to investigate whether empowerment provides them the agency to avoid exceeding their own reproductive goals despite this covert pressure. Of the total sample, 5,914 women met these criteria. However, due to missing data on some explanatory variables, the final analytical sample used in this study comprises 5,855 married or cohabiting women. A participant flow diagram is presented in Fig. 1.

We merged multi-phase DHS datasets (Table 1) as data collection years varied across countries. To ensure comparability, we retained only common variables shared by all nations. To align with recent trends, we included only post-2010 data, excluding Somalia (no data) and South Sudan (1989–1990).

**Table 1:** The member states of the EAC, their respective years of collection of DHS data and their respective phases

Country	Year of collection	Phase
Burundi	2015–2016	7
DRC*	2013–2014	6
Kenya	2022	8
Rwanda	2019	7
Somalia	No data	-
South Sudan	1989–1990	1
Tanzania	2022	8
Uganda	2016	7

*Democratic Republic of the Congo

The methodology of data collection and variable construction is well explained on the DHS program’s official website [46].

### Variables

#### Dependent variables

Based on our study aim, the primary dependent variable is a dichotomous measure we named, “woman remained within her ideal family size” which indicates whether a woman has stayed within her ideal number of children. This variable is not part of the standard DHS dataset, but it is constructed from original DHS variables (detailed below).

The dependent variable is obtained by subtracting the woman’s ideal number of children (V613) and the number of living children (V218). This was mathematically operationalized as:

- remained within ideal family size (coded as 1): (Ideal number − Living children) ≥ 0;
- Exceeded ideal family size (coded as 0): (Ideal number − Living children) < 0.

This construction follows previous DHS-based studies that have compared women’s ideal number of children (V613) with their actual living children (V218) to examine fertility preferences and outcomes [34, 36, 37, 47]. We chose this approach due to its high relevance to the study’s context, where remaining within desired family size is a key reproductive health outcome in high-fertility settings such as the EAC.

In the dataset, some women provided non-numeric responses about their ideal number of children (INC), such as “up to God/as Allah wills,” “as many as possible,” “as many as I can care for,” or “any number.” To minimize bias, previous studies [34–36] have judged the right to use the mean INC of women with numeric responses as a substitute for non-numeric responses. In this study, we adopt a similar approach, but we prefer using country-level medians over means to reduce the influence of outliers.

#### Independent variables

We use an empowerment index developed in 2017 based on questions from DHS datasets [44]. Initially designed to assess empowerment in African countries, it was adapted for global use to enable cross-country comparisons among developing nations. It is called the “survey-based women’s empowerment” index, abbreviated as the “SWPER” index and pronounced as SUPER, as suggested by the authors [44]. This index analyzes empowerment as a health outcome or determinant through three domains: attitude toward violence, social independence, and decision-making. The original SWPER index consists of 15 questions [43], but in the global one used in this study, slight modifications were applied, and one question was removed from the original version. The SWPER index was constructed using the sampling weights provided by Ewerling et al. [44]. As this study focuses on associations rather than nationally representative prevalence estimates, additional weighting beyond the SWPER construction was not applied and is unlikely to materially affect the results. Following the SWPER index guidelines [43, 44], each domain was classified into three empowerment levels using the following thresholds: for Attitude to violence, low empowerment corresponds to scores ≤ -0.700, medium empowerment to scores > -0.700 and ≤ 0.400, and high empowerment to scores > 0.400. For Social independence, low empowerment is defined as ≤ -0.559, medium as > -0.559 and ≤ 0.293, and high as > 0.293. For Decision-making, low empowerment corresponds to ≤ -1.000, medium to > -1.000 and ≤ 0.600, and high to > 0.600. The questions used to calculate the SWPER index are provided below. The formulas, the steps for calculating the SWPER scores for empowerment levels are provided in the appendix of the original articles [43, 44]. 

The variables included in the SWPER-global empowerment index as provided by Ewerling et al. [43]:

- Justification of wife-beating if she goes out without telling her husband.
- Justification of wife-beating if she neglects the children.
- Justification of wife-beating if she argues with her husband.
- Justification of wife-beating if she refuses to have sex with her husband.
- Justification of wife-beating if she burns the food.
- Frequency of reading newspaper or magazine.
- Woman education.
- Age of respondent at cohabitation.
- Age of respondent at first birth.
- Age difference: woman’s minus husband’s age.
- Education difference: woman’s minus husband’s years of schooling.
- Who usually decides on the respondent’s health care?
- Who usually decides on large household purchases?
- Who usually decides on visits to family or relatives?

#### Control variables

Taking into account the variables included in the SWPER index, which is our primary independent variable [43, 44], and based on our review of previous research [34–37], as well as on available variables in the DHS datasets [45], as potential confounders, we include sociodemographic and economic such as the woman’s age in 5-year groups (V013), the type of place of residence (V025), the literacy level (V155), the wealth index (V190), the distance to health facility (V467D), and the number of household members (V136). These variables could influence the woman’s ideal number of children and whether she remains within her ideal number. Most of these variables originate from the DHS; others are renamed and recoded based on the original ones. The following are the recoded variables:

- “Literacy level” is recoded as “ability to read a sentence” and has three values: cannot read, can read partly, and can read whole.
- “Getting medical help for self: distance to a health facility” is renamed “Distance to a health facility.” From the DHS coding “no problem,” “big problem,” and “not a big problem,” we created a binary variable removing “no problem” due to missing response data.
- “Number of household members” is originally a continuous variable. We recoded it into a group variable and renamed it “grouped household size”. We did not find any universal threshold for classifying the household size groups. Previous authors [35, 37, 48, 49] adapted their classification based on their study area and cultural context; we opted for the classification used in this study done in Rwanda [48] based on the fact that Rwanda is a country member state of the EAC, which can give a closer picture of the household size in that side of Africa. We therefore used “grouped household size” as our new variable and classified it into small < 5, medium: 5–6, medium-large: 7–9, and large: 10+.

### Statistical analysis

This study used IBM SPSS Statistics 24 (IBM Corporation, Armonk, NY, USA). Firstly, a general description of the dependent and control variables was conducted. The frequencies and percentages were calculated for categorical variables, while medians and means were computed for continuous variables.

Then, following the steps as described in the original articles by Ewerling et al. [43, 44], empowerment levels as the primary independent variable were calculated and classified into low, medium, and high levels. To standardize the SWPER score, we used the mean and standard deviation specific for the Eastern and Southern African regions [43]. We chose this regional standardization because five of the six countries included in our study belong to this region, making it the most appropriate reference population for our analysis [43]. Given that the dependent variable is binary (indicating whether the woman has remained within ideal number of children.), we opted for binary logistic regression. Ordinal predictors were coded as integer values (1, 2, 3, etc.) and entered into the logistic regression model as a single numeric predictor (linear term), thereby modeling a monotonic linear trend across categories. All models are adjusted for country fixed effects, with Burundi as the reference category.

First, we conducted univariable analyses to examine the relationship between:

- Each empowerment domain and the dependent variable and.
- Each covariate and the dependent variable.

Odds ratios (OR) with 95% Confidence Interval (CI) and corresponding *p*-values were computed.

The set of candidate variables for the multivariable binary logistic regression model was defined based on two criteria: (1) variables that were statistically significant in univariable analyses (*p* < 0.05), and (2) variables identified in the literature as clinically relevant predictors of the outcome. In total, nine candidate variables were considered for inclusion in the multivariable model.

Variable selection was performed using LASSO (Least Absolute Shrinkage and Selection Operator) regularization, which allows for simultaneous variable selection and coefficient shrinkage. The optimal penalty parameter (λ) was determined using cross-validation. Consequently, only the most informative predictors were retained in the final model.

Prior to running the multivariable model, we assessed included variables for potential multicollinearity using Variance Inflation Factor (VIF), with values greater than 5 indicating potential multicollinearity. In the multivariable logistic regression model the Adjusted Odds Ratios (AORs) were calculated. A *p*-value of less than 0.05 was considered statistically significant. A graphical presentation of the multivariable logistic regression model was generated using the Forester Package in R. Cases with missing data on any variable were excluded from the analysis (listwise deletion).

Taking into account the fact that some women may have not yet fully completed their fertility for instance the group of the 35–39 years old, we conducted sensitivity analysis restricting the sample to women who had either met their ideal number of children exactly or exceeded it (i.e., excluding those with living children < ideal number).

The analysis syntax used for statistical analyses is provided in the Supplementary File.

## Results

### Study population characteristics and findings from the univariable model

This study includes 5855 participants who have complete data for all variables. Missing data proportions were low (< 3%) for all variables included in the analysis. As presented in Tables 2 and 3, among the 5855 married/cohabiting women aged 35 and above who believe their husbands want more children than they do, nearly two-thirds (63.3%) did not exceed their ideal number of children. The pooled median ideal number of children across EAC countries (based on survey years ranging from 2013 to 2022) is 5, with values ranging from 0 to 20 (Supplementary Table 1). 

**Table 2:** Description of independent variables and their statistical significance based on univariable analysis

		Remained within ideal family size			
Variables	Total: *n* = 5855 (100.0%)	Yes: *n* = 3709 (63.3%)	No: *n* = 2146 (36.7%)	OR (95%CI)	*p*-value
Emp. Attitude Toward Violence					
Low	1225 (21.3%)	797 (22.0%)	428 (20.3%)		*0.440*
Medium	3511 (61.1%)	2170 (59.8%)	1341 (63.3%)	0.96 (0.88–1.06)	
High	1011 (17.6%)	661 (18.2%)	350 (16.5%)		
Emp Social Independence					
Low	734 (12.8%)	436 (12.0%)	298 (14.1%)		*< 0.001*
Medium	2670 (46.5%)	1573 (43.4%)	1097 (51.8%)	1.41 (1.29–1.53)	
High	2343 (40.8%)	1619 (46.5%)	724 (34.2%)		
Emp. Decision-making					
Low	722 (12.6%)	497 (13.7%)	225 (10.6%)		*< 0 0.001*
Medium	3432 (59.7%)	2186 (60.3%)	1246 (58.8%)	0.88 (0.80–0.97)	
High	1593 (27.7%)	945 (26.0%)	648 (30.6%)		

*Emp* empowerment

**Table 3:** Description of control variables and their statistical significance based on univariable analysis

		Remained within ideal family size			
Variables	Total: *n* = 5855 (100.0%)	Yes: *n* = 3709 (63.3%)	No: *n* = 2146 (36.7%)	OR (95%CI)	*p*-value
Age in 5 years groups					
35–39	2565 (43.8%)	1709 (46.1%)	856 (39.9%)		*< 0.001*
40–44	1874 (32.0%)	1168 (31.5%)	706 (32.9%)	0.83 (0.78–0.89)	
45–49	1416 (24.2%)	832 (22.4%)	584 (27.2%)		
Type of place of residence					
Urban*	1390 (23.6%)	934 (25.2%)	456 (21.2%)	0.94 (0.82–1.07)	*0.360*
Rural	4465 (76.4%)	2775 (74.8%)	1690 (78.8%)		
Household size grouped					
Smaller: <5	1247 (21.3%)	1062 (28.6%)	185 (8.6%)		*< 0 0.001*
Medium: 5–6	1762 (30.0%)	1204 (32.5%)	558 (26.0%)	0.49 (0.46–0.53)	
Medium-large: 7–9	2090 (35.8%)	1074 (29.0%)	1016 (47.3%)		
Large: 10+	756 (12.9%)	369 (9.9%)	387 (18.0%)		
Wealth index					
Poorest	1338 (22.9%)	879 (23.7%)	459 (21.4%)		*0.840*
Poorer	1190 (20.4%)	743 (20.0%)	447 (20.8%)		
Middle	1278 (21.8%)	783 (21.1%)	495(23.1%)	1.04 (0.96–1.05)	
Richer	1117 (19.0%)	690 (18.6%)	427(19.9%)		
Richest	932 (15.8%)	614 (16.6%)	318 (14.8%)		
Distance to a health facility					
Big problem*	2299 (39.4%)	1447 (39.0%)	852 (39.7%)	1.03 (0.98–1.24)	*0.090*
Not a big problem	3556 (60.6%)	2262 (61.0%)	1294 (60.3%)		
Ability to read a sentence					
Cannot	2520 (43.6%)	1642 (44.5%)	878 (41.3%)	0.91 (0.86–0.97)	*0.002*
Read partly	562 (9.6%)	328 (8.9%)	234 (11.0%)		
Read whole	2737 (46.8%)	1722 (46.6%)	1015 (47.7%)		

*Reference group

#### Findings on women’s empowerment

We assessed women’s empowerment across three domains: social independence, decision-making, and attitude toward violence. Most women fall into the medium empowerment level (61.1%, 46.5%, and 59.7%, respectively, for attitude toward violence, social independence, and decision-making). The domain of attitude toward violence has the highest proportions in the low empowerment level (21.3%), indicating that women are more empowered in social independence and decision-making (Table 2). 

Although in the domain of attitude toward violence, greater percentages are noticed among those who have reached their ideal number and those who did not in the medium level of empowerment (Table 2), those with low and high empowerment levels are the ones who have mainly remained within their ideal number of children with very close proportions (65.1% vs. 65.4%) (Supplementary Table 2).

In the domain of social independence, most of those who remained within their ideal number came from the higher empowerment level, while those who did not primarily came from the medium level (Table 2). However, when comparing subgroups by empowerment levels, the highest rate of non-excess (69.1%) was observed among women in the highest empowerment level (Supplementary Table 2).

In the decision-making domain, the pathway is similar to that of the attitude toward violence, where greater percentages of those who remained within their goal and those who did not are observed in the medium level of empowerment (Table 2). However, comparing each subset of each level, most of those who did not exceed their goal belong to the low level of empowerment (68.8%). In comparison, most of those with the highest empowerment level did not remain within their ideal number of children (40.7%) (Supplementary Table 2).

Concerning the findings from the univariable model, the three empowerment domains show distinct paths. Two domains are significantly associated to the non-excess of the woman’s ideal number of children and that in opposite directions. The domain of social independance shows a protective association (OR = 1.41, 95% CI:1.29–1.53) while the decision making one reveals a negative effect (OR = 0.88, 95% CI : 0.80–0.97). The domain of attitude toward violence has a non-significant effect (OR = 0.96, 95% CI: 0.88–1.06).

#### Findings on control variables 

Analyzing those who remained within their goal and those who did not in general, the majority in both outcomes (Yes/No) belong to the age group of 35–39 years old (43.8%, 39.9%, respectively) (Table 3); but the subgroup comparisons inside each age group show that the women 35–39 are the most to remain within their goal (66.6%) while those 44–49 are the least do so (58.8%) (Supplementary Table 3). The mean age is 40.89 ± 4.25.

Seventy-six point four percent of the women in this study reside in rural areas (Table 3). The findings show that more women in urban areas have remained within the goal of having the desired number of children (67.2%) than those from rural areas (62.2%) (Supplementary Table 3).

Most (60.6%) of these women state that the distance to the health facility is not a big problem (Table 3). When it comes to staying within the ideal number of children, only a 0.7% difference is observed between those who perceive the distance to the health facility as a big problem (63.6%) and those who perceive it as not a big problem (62.9%) (Supplementary Table 3).

Most households (35.8%) have 7–9 members, followed by the group with 5–6 members (30%) (Table 3). Most of those who remained within their goal are in small households (85.2%). The largest number of those who exceeded their goal is in large households (51.2%) (Supplementary Table 3).

There are no big differences regarding the wealth index; the smallest proportion (15%) is in the wealthiest quintile, and the remaining quintiles range between 19% and 22% (Table 3). However, remaining within the ideal number of children is highest among the richest (65.9%) and the poorest (65.7%) (Supplementary Table 3).

The proportion of women who can read a whole sentence (46.8%) and those who cannot read (43.6%) is almost equal (Table 3). Similarly, these groups show similar results in remaining within their ideal number of children (62.9% vs. 65.2%, respectively) (Supplementary Table 3).

According to the findings illustrated in Table 3, age, household size and literacy are the only control variables in the univariable model which are significantly associated with remaining within one’s ideal number of children. All three express a negative association with the outcome. The other three control variables: type of place of residence (OR = 0.94, *p* = 0.36), wealth index (OR = 1.04, *p* = 0.84), and distance to health facility (OR = 1.03, *p* = 0.09) are non-significant in this model.

### Multivariable binary logistic regression

**Fig. 2:**
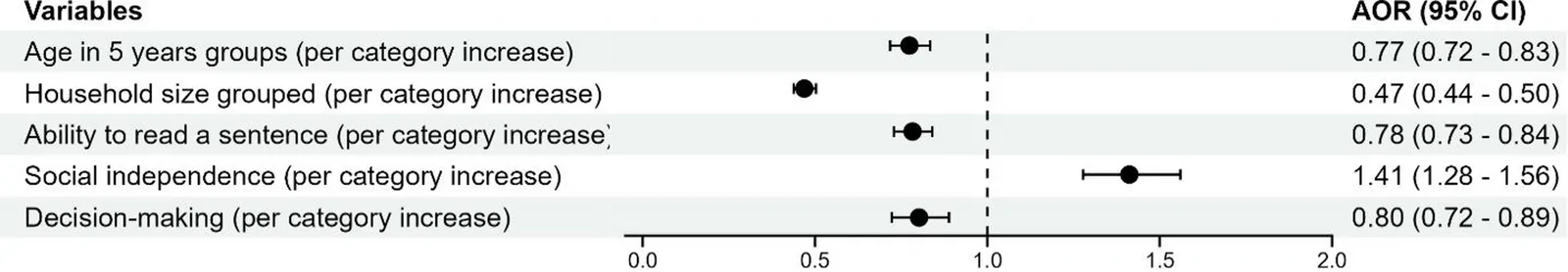
Graphical presentation of the multivariable binary logistic regression model

The multivariable logistic regression model includes all variables significant at the univariable level and identifies factors independently associated with a woman remaining within her ideal number of children. This model is adjusted for age, household size, literacy and two SWPER empowerment domains, as one domain is not significant in the univariable model. No multicollinearity is detected between variables in our model, all VIF values are below 5 (maximum VIF = 1.22), confirming the absence of multicollinearity. The VIF values for all predictors are available in the supplementary Table 4. The VIF values for all predictors are available in the supplementary Table 4. Model performance was supported by a significant omnibus test of model coefficients (*p* < 0.001) and a Nagelkerke pseudo R² of 0.245. The results of the adjusted analysis are presented below in Fig. 2. In this model, several key associations between explanatory variables and the outcome variable are observed:

- Association with SWPER empowerment domains (the independent variable): two empowerment domains are significant predictors, but in contrasting directions. A higher level of social independence is associated with a 59% increase in the odds of remaining within one's ideal number of children (AOR = (1.41;95%CI 1.28-1.56)). Conversely, a higher level of decision-making power is associated with a 20% reduction in the odds of remaining within this goal (AOR = (0.80; 95% CI: 0.72-0.89)). The attitude toward violence domain is not included in this model, as it was not significantly associated with the outcome in the univariable model (OR=0.96; 95% CI 0.88-1.06).
- Association with control variables: Consistent with the significance in the univariable model, these variables are also significant in the multivariable model, confirming that all the control variables are independently associated with the outcome: Being in a higher age group reduces the odds of remaining within the ideal number by 23%. Being able to read a sentence is associated with 22% lower odds of not exceeding their ideal number of children, and a larger household size reduces the chances by 53%.

In sensitivity analysis restricted to women who had met their ideal exactly or exceeded it (*n* = 2,967), social independence remained associated with remaining within ideal family size (OR = 1.43, 95% CI: 1.23–1.65), while decision-making empowerment was no longer significant. Age, ability to read a sentence and household size demonstrated clear dose-response patterns.

## Discussion

Our results have shown that considering our primary independent variable, women’s empowerment, only two domains of the SWPER index are significantly associated with remaining within the woman’s ideal number of children. The domain of attitude toward violence showed no significant association with remaining within the woman’s goal. From the control variables, age, ability to read a sentence, and household size are linked to remaining within the ideal number of children among women who perceive that their partner wants more children than they do. These findings are statistically significant both in the univariable and multivariable models (Tables 2 and 3; Fig. 2).

Exceptionally, only the empowerment domain of social independence is positively associated with the outcome, while all the other variables are negatively linked (Fig. 2).

The higher the level of empowerment in social independence, the higher the likelihood of the woman remaining within her ideal number of children. This could be a result of the nature of the items that constitute the social independence SWPER domain. This domain takes into account the woman’s education level, her education relative to her husband, exposure to media and information, and life-course factors such as age at first cohabitation and age at first birth [43, 44]. A woman with higher empowerment levels of social independence based on SWPER reflects then a more educated and better informed woman among other traits. These characteristics enhance her autonomy and ability to make informed reproductive decisions including those that will allow her to remain within her ideal number of children. For instance, socially independent women are more likely to have access to contraceptives and to use them in case they have reached their ideal number of children [33, 44, 50]. However, the findings of our study are in contrast to those found in Ghana, where women’s empowerment measured using the SWPER index also shows that those with high social-independence empowerment had lower chances of remaining within their desired number of children [35]. The possible explanation for this difference could be country variations and cultural differences since Ghana is a West African country, whereas our study focuses on East African countries. Different countries and regions also mean different healthcare systems; perhaps there are childbearing-friendly policies in one region, as in Burundi, where women and children under 5 years old have free healthcare [51].

Our findings concerning the decision making domain reveal a critical paradox: while women with higher decision making empowerment are assumed to have greater control over their reproductive lives and hence remain within their ideal number of children, we found that this expectation is less likely to happen. In the specific context of this study, we suggest that one of the main causes of these counterintuitive findings is the composition of the used index itself. The SWPER index, although a proven valuable tool for measuring women’s empowerment in developing countries, did not include questions concerning reproductive decisions, such as the woman’s power in bargaining sex or contraception decision-making; in other words, sexual agency is not deeply explored. The second reason likely reflects the cross-sectional nature of DHS data which does not allow us to determine the temporal direction of this relationship. A Reverse causality is possible. Since these women are assumed to have reached their reproductive timeline limit, the decision making empowerment they have may be in reality granting them the authority to manage the consequences of high fertility rather than the power to prevent it. This may mean that, the women gain decision-making power within marriage precisely by successfully embodying the role of a fertile mother. Hence, this empowerment in decision making in this specific study might signify a woman has had many children (with a possibility of having surpassed her ideal number of children) and, as a result, has gained the respect that allows her to make other decisions. Similar patterns have been documented in earlier research, such as the studies by Acharya et al. [52] and Mustafiz et al. [53], where higher parity was associated with increased decision-making autonomy.

On the other hand, this study is not the first to find counterintuitive findings when it comes to the relationship between decision making empowerment and fertility outcomes in the context of having more children than desired. Haque et al. [34] and Ushma and Karasek [36] have found that a woman’s power in decision making is associated with having more children than she initially ideally desired.

Other studies [35, 37] yielded findings that are contrary to our results showing that having power in decision-making is associated with remaining within the ideal number of children, which contradicts our findings. This is in accord with prior literature [33, 42, 54–57] that has evidenced that greater empowerment in decision-making is associated with having a say in fertility decisions and their overall reproductive health.

According to our findings in Fig. 2, the higher the age group, the lower the odds of the woman remaining within her ideal number of children. A similar pattern is observed in Guinea and Zambia, where older age was associated with a higher likelihood of exceeding desired fertility [36]. This is because the older a woman gets, the closer she gets to ending her childbearing years. Moreover, high age is associated with unintended pregnancies [23, 58].

Our findings reveal that the larger the household, the lower the chances of remaining within the ideal number of children. These findings are in line with those found in Morocco, where women with larger family sizes, specifically those with more than six children, were more likely to have more children than they desire [27]. This is evident because if these women have large families, this implies that they have most likely already exceeded their ideal number of children, suggesting limited reproductive control. Such circumstances reduce their ability to assert their preferences, particularly when they perceive their husbands desire more children. This could also portray a lack of reproductive empowerment, limited access to family planning, and an unsupportive environment for avoiding exceeding the desired fertility outcome for women. Moreover, there is a possibility that these women are genuinely enjoying large families, especially in settings where child labor is prevalent. However, our study findings contradict those from other African contexts that explored whether women’s empowerment level influences their ability to have desired children. They found that large households were more likely to not exceed the desired number of children [35, 37]. This discrepancy may result from our study’s focus on the subgroup of women who perceive that their husbands want more children than they do or could result from regional variations.

Literacy, defined as the ability to read a sentence, was negatively associated with remaining within ideal family size (OR = 0.78; 95% CI: 0.73–0.84). These findings appear to be paradoxical given that women’s literacy and education are expected to be positively associated with fertility outcome according to previous literature [43, 44, 59, 60]. However, the distribution of literacy within the study population may offer a clearer explanation: nearly half of the women were unable to read (43%), and more than three-quarters resided in rural areas (76%). In predominantly rural contexts, the ability to read may not necessarily translate into improved access to reproductive health services, stronger decision-making empowerment, or effective contraceptive use. In the high-fertility EAC settings, these findings indicate that basic literacy does not translate into household bargaining power, particularly when husbands desire more children.

Several studies have shown mixed associations and variations between empowerment and fertility [33, 34, 61, 62]. This suggests that the relationship is complex and that more external factors could influence it.

The fact that decision-making empowerment lost significance after excluding women below their ideal number suggests its main effect may be partly driven by women with incomplete fertility. 

### Policy and programmatic recommendations

Given that social independence empowerment is the only variable positively associated with remaining within the ideal number of children for women in the current study, we recommend that concerned stakeholders, such as ministries of gender, of education, and/or of health of each respective EAC country; together with Non-Governmental Organizations (NGOs) should prioritize and fund programs that can enhance women’s social independence. For instance, community dialogues that challenge norms restricting women’s mobility and participation in social life outside the home.

Concerning the findings from other variables, we recommend: careful attention concerning family support policy design, particularly regarding how benefit structures and eligibility criteria may influence household decisions about family size; reinforcing the existing provision of free modern contraceptive methods accompanied by free, accessible, and confidential consultations on reproductive health options; reducing rural areas through more urbanization would improve social exposition. 

### Study strengths and limitations

To the best of our knowledge, this is the first study to analyze the link between women’s empowerment and remaining within the ideal number of children, including the women’s perception of her husband’s desired family size in the countries of the EAC. The strengths of our study are, first, the use of nationally representative data; with this, our findings reflect a country-level picture. The focus on the EAC is another strength. We opted for this African region due to its free population mobility within the community and shared common language (Swahili) [63]. This may generate a blending of cultures and social norms between the member states of this community. The fact that we have used a standardized empowerment measure provides reliable results and facilitates comparability.

This study has three main limitations: First, two countries from the EAC were not included due to unavailable or very old data. Second, merging data required excluding variables, like religion and health insurance coverage (potential confounders), that were not common to all the countries. Third, we did not adjust for the clustered sampling design of the DHS. While clustering is unlikely to bias point estimates, it may lead to underestimated standard errors. Given our focus on associations rather than prevalence, and the inclusion of country fixed effects, the impact on our main findings is likely minimal.

This study relied exclusively on women’s self-reported data. Future research incorporating men’s perspectives or couple-level data could provide a more holistic understanding of fertility intentions and empowerment dynamics in the EAC. Including both partners may reveal areas of agreement or disagreement within couples, and would help better understanding the woman’s answers by also considering her husband’s perspective.

## Conclusions

This study highlights the complex relationship between women’s empowerment and fertility outcomes in the East African Community. Using nationally representative DHS data, we focused on women who perceive their husbands want more children than they do. Employing the SWPER empowerment index and controlling for key confounders, including age, residence type, household size, wealth, literacy, and distance to health facilities, we assessed how empowerment across three domains (attitude toward violence, social independence, and decision-making) influences remaining within fertility goals. Contrast outcomes where social-independence empowerment was positively linked to remaining within the ideal number of children and decision-making empowerment was negatively linked to the same outcome. On the other hand, the attitude toward violence domain of the SWPER empowerment index showed no significant association, confirming the multidimensionality and context-dependent nature of women’s empowerment and its relationship with fertility outcomes.

Based on these findings, women’s autonomy and reproductive agency should be strengthened in the East African Community through policy and actionable interventions. Concerned governments and stakeholders in the EAC recommend empowerment strategies targeting primarily the factors that enhance social-independence empowerment, couple-focused interventions, and easy access to and promotion of the usage of reproductive healthcare services to help women exercise reproductive rights and accomplish their family-size goals.

## Supplementary Information

Supplementary Material 1.

## Data Availability

This study used Demographic and health survey data from the countries involved in this study. The data are openly available for any researcher at [https://www.dhsprogram.com/data/] (https:/www.dhsprogram.com/data).

## Abbreviations

SPSS: Statistical Package for the Social Sciences
SWPER: Survey-based Women’s emPowERment
SDGs: Sustainable Development Goals
EAC: East African Community
DHS: Demographic and Health Survey
DRC: Democratic Republic of Congo
INC: Ideal number of children
OR: Odds Ratio
CI: Confidence Interval
AOR: Adjusted Odds Ratio
VIF: Variance Inflation Factor
