# Supplementary material for: Women’s empowerment and remaining within ideal family size in the East African Community
Source: Reprod Health. 2026 Jul 24;23:187. doi: 10.1186/s12978-026-02412-y (PMC13681907; doi:10.1186/s12978-026-02412-y)
Supplement: Supplementary file 1 [file 12978_2026_2412_MOESM1_ESM.docx]

**Women's empowerment and remaining within ideal family size in the East African Community**

AKIMANA Ange Chanelle^1^, Bukumiric Zoran^2^^,3^, KANA Didace^4⌧^^ORCID^, Vukovic Dejana^5^

Supplementary Table 1: Median women’s ideal number of children by country and in the EAC

| Country | Median | Range |
| --- | --- | --- |
| Burundi | 4 | 0-15 |
| DRC | 7 | 0-20 |
| Kenya | 4 | 0-20 |
| Rwanda | 4 | 0-12 |
| Tanzania | 6 | 0-20 |
| Uganda | 6 | 0-20 |
| EAC | 5 | 0-20 |

Supplementary Table 2. Description of the SWPER index domains and their statistical significance based on univariate analysis: subgroup-specific results.

|  |  | ***Remained within ideal family size*** | |  |  |
| --- | --- | --- | --- | --- | --- |
| Variables | Total: n=5855 (100.0%) | Yes: n=3709  (63.3%) | No: n=2146  (36.7%) | OR (95%CI) | p-value |
| Emp. Attitute Toward violence  Low  Medium  High | 1225  3511  1011 | 797 (65.1%)  2170 (61.8%)  661 (65.4%) | 428 (34.9%)  1341 (38.2%)  350 (34.6%) | 0.96 (0.88-1.06) | *0.440* |
| Emp Social Independence  Low  Medium  High | 734  2670  2343 | 436 (59.4%)  1573 (58.9%)  1619 (69.1%) | 298 (40.6%)  1097 (41.1%)  724 (30.9%) | 1.41 (1.29-1.53) | *< 0.001* |
| Emp. Decision-making  Low  Medium  High | 722  3432  1593 | 497 (68.8%)  2186 (63.7%)  945 (59.3%) | 225 (31,2%)  1246 (36.3%)  648 (40.7%) | 0.88 (0.80-0.97) | *<0.001* |

Emp= empowerment

Supplementary Table 3. Description of control variables and their statistical significance based on univariable analysis: subgroup-specific results.

|  |  | ***Remained within ideal family size*** | |  |  |
| --- | --- | --- | --- | --- | --- |
| Variables | Total: n=5855 (100.0%) | Yes: n=3709  (63.3%) | No: n=2146  (36.7%) | OR (95%CI) | *p-value* |
| Age in 5 years groups  35-39  40-44  45-49 | 2565  1874  1416 | 1709 (66.6%)  1168 (62.3%)  832 (58.8%) | 856 (33.4%)  706 (37.7%)  584 (41.2%) | 0.83 (0.78-0.89) | *< 0.001* |
| Type of place of residence  Urban*  Rural | 1390  4465 | 934 (67.2%)  2775 (62.2%) | 456 (32.8%)  1690 (37.8%) | 0.94 (0.82-1.07) | *0.360* |
| Household size grouped  Smaller: <5  Medium: 5-6  Medium-large: 7-9  Large: 10+ | 1247  1762  2090  756 | 1062 (85.2%)  1204 (68.3%)  1074 (51.4%)  369 (48.8%) | 185 (14.8%)  558 (31.7%)  1016 (48.6%)  387 (51.2%) | 0.49 (0.46-0.53) | *< 0.001* |
| Wealth index  Poorest  Poorer  Middle  Richer Richest | 1338  1190  1278  1117  932 | 879 (65.7%)  743 (62.4%)  783 (61.3%)  690 (61.8%)  614 (65.9%) | 459 (34.3%)  447 (37.6%)  495(38.7%)  427(38.2%)  318 (34.1%) | 1.04 (0.96-1.05) | *0 .840* |
| Distance to health facility  Big problem *  Not a big problem | 2299  3556 | 1447 (62.9%)  2262 (63.6%) | 852 (37.1%)  1294 (36.4%) | 1.03 (0.98-1.24) | *0 .090* |
| Ability to read a sentence  Cannot  Read partly  Read whole | 2520  562  2737 | 1642 (65.2%)  328 (58.4%)  1722 (62.9%) | 878 (34.8%)  234 (41.6%)  1015 (37.1%) | 0.91 (0.86-0.97) | *0.002* |

*Reference group

Supplementary Table 4: Predictors’ VIF values

| Predictors | VIF values |
| --- | --- |
| Age in five years groups | 1.010 |
| Household size grouped | 1.061 |
| Ability to read a sentence | 1.214 |
| Social Independence Empowerment | 1.223 |
| Decision- making empowerment | 1.043 |

Analysis syntax

**Descriptives statistics**

```
CROSSTABS
  /TABLES=V013Age.in.5year.groups V025Type.of.place.of.residence V190Wealth.index.combined
    Household.size_Grouped V467DDistance.To.Health.Facility Ability.to.read.a.sentence Outcome_ESA_Emp_att
    Outcome_ESA_Emp_Soc Outcome_ESA_Emp_Dec BY YesNo_remained.within.ideal.family.size
  /FORMAT=AVALUE TABLES
  /CELLS=COUNT COLUMN
  /COUNT ROUND CELL.
```

**Univariate logistic regression**

1. LOGISTIC REGRESSION VARIABLES YesNo_remained.within.ideal.family.size

/METHOD=ENTER V013Age.in.5year.groups Countries

/CONTRAST (Countries)=Indicator(1)

/PRINT=CI(95)

/CRITERIA=PIN(0.05) POUT(0.10) ITERATE(20) CUT(0.5).

2. LOGISTIC REGRESSION VARIABLES YesNo_remained.within.ideal.family.size

/METHOD=ENTER V025Type.of.place.of.residence Countries

/CONTRAST (Countries)=Indicator(1)

/PRINT=CI(95)

/CRITERIA=PIN(0.05) POUT(0.10) ITERATE(20) CUT(0.5).

3. LOGISTIC REGRESSION VARIABLES YesNo_remained.within.ideal.family.size

/METHOD=ENTER Household.size_Grouped Countries

/CONTRAST (Countries)=Indicator(1)

/PRINT=CI(95)

/CRITERIA=PIN(0.05) POUT(0.10) ITERATE(20) CUT(0.5).

4. LOGISTIC REGRESSION VARIABLES YesNo_remained.within.ideal.family.size

/METHOD=ENTER V190Wealth.index.combined Countries

/CONTRAST (Countries)=Indicator(1)

/PRINT=CI(95)

/CRITERIA=PIN(0.05) POUT(0.10) ITERATE(20) CUT(0.5).

5. LOGISTIC REGRESSION VARIABLES YesNo_remained.within.ideal.family.size

/METHOD=ENTER V467D_Distance.To.Health.Facility Countries

/CONTRAST (Countries)=Indicator(1)

/PRINT=CI(95)

/CRITERIA=PIN(0.05) POUT(0.10) ITERATE(20) CUT(0.5).

6. LOGISTIC REGRESSION VARIABLES YesNo_remained.within.ideal.family.size

/METHOD=ENTER Ability.to.read.a.sentence Countries

/CONTRAST (Countries)=Indicator(1)

/PRINT=CI(95)

/CRITERIA=PIN(0.05) POUT(0.10) ITERATE(20) CUT(0.5).

7. LOGISTIC REGRESSION VARIABLES YesNo_remained.within.ideal.family.size

/METHOD=ENTER Outcome_ESA_Emp_att Countries

/CONTRAST (Countries)=Indicator(1)

/PRINT=CI(95)

/CRITERIA=PIN(0.05) POUT(0.10) ITERATE(20) CUT(0.5).

8. LOGISTIC REGRESSION VARIABLES YesNo_remained.within.ideal.family.size

/METHOD=ENTER Outcome_ESA_Emp_Soc Countries

/CONTRAST (Countries)=Indicator(1)

/PRINT=CI(95)

/CRITERIA=PIN(0.05) POUT(0.10) ITERATE(20) CUT(0.5).

9. LOGISTIC REGRESSION VARIABLES YesNo_remained.within.ideal.family.size

/METHOD=ENTER Outcome_ESA_Emp_Dec Countries

/CONTRAST (Countries)=Indicator(1)

/PRINT=CI(95)

/CRITERIA=PIN(0.05) POUT(0.10) ITERATE(20) CUT(0.5).

**Multicollinearity check**

```
REGRESSION
  /MISSING LISTWISE
  /STATISTICS COEFF OUTS R ANOVA COLLIN TOL
  /CRITERIA=PIN(.05) POUT(.10)
  /NOORIGIN
  /DEPENDENT YesNo_remained.within.ideal.family.size
  /METHOD=ENTER V013Age.in.5year.groups Household.size_Grouped Ability.to.read.a.sentence
    Outcome_ESA_Emp_Soc Outcome_ESA_Emp_Dec dummy.Countries_Congo dummy.Countries_Kenya
    dummy.Countries_Rwanda dummy.Countries_Tanzania dummy.Countries_Uganda.


Multivariate logistic regression
LOGISTIC REGRESSION VARIABLES YesNo_remained.within.ideal.family.size
  /METHOD=ENTER V013Age.in.5year.groups Household.size_Grouped Ability.to.read.a.sentence
    Outcome_ESA_Emp_Soc Outcome_ESA_Emp_Dec Countries
  /CONTRAST (Countries)=Indicator(1)
  /PRINT=CI(95)
  /CRITERIA=PIN(0.05) POUT(0.10) ITERATE(20) CUT(0.5).

Sensitivity analyses
```

1. SORT CASES BY YesNo_remained.within.ideal.family.size_nominal.

```
SPLIT FILE SEPARATE BY YesNo_ remained.within.ideal.family.size_nominal.
LOGISTIC REGRESSION VARIABLES YesNo_ remained.within.ideal.family.size
/METHOD=ENTER dummy.Countries_Congo dummy.Countries_Kenya dummy.Countries_Rwanda
dummy.Countries_Tanzania dummy.Countries_Uganda V013Age.in.5year.groups Household.size_Grouped
  Ability.to.read.a.sentence Outcome_ESA_Emp_Soc Outcome_ESA_Emp_Dec
  /PRINT=CI(95)
  /CRITERIA=PIN(0.05) POUT(0.10) ITERATE(20) CUT(0.5).
```

2. SPLIT FILE SEPARATE BY YesNo_remained.within.ideal.family.size

LOGISTIC REGRESSION VARIABLES YesNo_ remained.within.ideal.family.size

/METHOD=ENTER V013Age.in.5year.groups Household.size_Grouped Ability.to.read.a.sentence

Outcome_ESA_Emp_Soc Outcome_ESA_Emp_Dec Countries

/CONTRAST (Countries)=Indicator(1)

/PRINT=CI(95)

/CRITERIA=PIN(0.05) POUT(0.10) ITERATE(20) CUT(0.5).
